# A national model of remote care for assessing and providing opioid agonist treatment during the COVID-19 pandemic: a report

**DOI:** 10.1186/s12954-020-00394-z

**Published:** 2020-07-17

**Authors:** Des Crowley, Ide Delargy

**Affiliations:** 1 Irish College of General Practitioners, Lincoln Place, Dublin 2, Ireland; 2 University College Dublin, Belfield, Dublin 4, Ireland

**Keywords:** Opioid agonist therapy, OAT, COVID-19, Opioid user, Remote health, Telemedicine

## Abstract

**Background:**

Health services globally are struggling to manage the impact of COVID-19. The existing global disease burden related to opioid use is significant. Particularly challenging groups include older drug users who are more vulnerable to the effects of COVID-19. Increasing access to safe and effective opioid agonist treatment (OAT) and other harm reduction services during this pandemic is critical to reduce risk. In response to COVID-19, healthcare is increasingly being delivered by telephone and video consultation, and this report describes the development of a national model of remote care to eliminate waiting lists and increase access to OAT in Ireland.

**Purpose and findings:**

The purpose of this initiative is to provide easy access to OAT by developing a model of remote assessment and ongoing care and eliminate existing national waiting lists. The Irish College of General Practitioners in conjunction with the National Health Service Executive office for Social Inclusion agreed a set of protocols to enable a system of remote consultation but still delivering OAT locally to people who use drugs.

This model was targeted at OAT services with existing waiting lists due to a shortage of specialist medical staff. The model involves an initial telephone assessment with COVID-risk triage, a single-patient visit to local services to provide a point of care drug screen and complete necessary documentation and remote video assessment and ongoing management by a GP addiction specialist. A secure national electronic health link system allows for the safe and timely delivery of scripts to a designated local community pharmacy.

**Conclusion:**

The development of a remote model of healthcare delivery allows for the reduction in transmission risks associated with COVID-19, increases access to OAT, reduces waiting times and minimises barriers to services. An evaluation of this model is ongoing and will be reported once completed. Fast adaptation of OAT delivery is critical to ensure access to and continuity of service delivery and minimise risk to our staff, patients and community. Innovative models of remote healthcare delivery adapted during the COVID-19 crisis may inform and have important benefits to our health system into the future.

## Introduction

Novel coronavirus disease (COVID-19) is a rapidly evolving pandemic that is threatening health systems worldwide. At the time of writing (9 April 2020), there are almost 1.5 million confirmed cases and over 85,000 deaths globally [1]. Older people, males and healthcare workers are disproportionally affected [2, 3].

Ireland like other European countries is struggling to contain this pandemic. The first case of COVID-19 was identified in Ireland at the end of February, and at the time of writing, there are 8089 confirmed cases and 263 COVID-related deaths [4]. Ireland was quick to respond to WHO guidance on the management of COVID-19 and implemented school and business closures at an early stage. Irish public health experts have expressed satisfaction at the public’s compliance with social distancing and self-isolation measures. These efforts appear to be flattening the curve with reduction in the rates of new infections [4]. However, concern has been expressed about the levels of and delays in testing and the capacity of our hospital and community services to cope with the expected surge in new cases.

The global burden of disease due to opioid use is increasing. Worldwide, there are an estimated 40.5 million people opioid dependant resulting in over 100,000 deaths annually [5]. Opioid users, in particular, people who inject opioids have increased physical and mental health care needs that make them particularly vulnerable during this pandemic [5, 6]. They are also at increased risk of homelessness and imprisonment; factors which impact negatively on their ability to social distance and self-isolate [6, 7]. There is an increased prevalence of HIV and hepatitis C infection among people who inject drugs further increasing their vulnerability [8]. To date there is no reports on the impact of COVID-19 on opioid users, but it is reasonable to expect that it will be significant.

It is recognised that opioid agonist treatment (OAT) is a safe and effective treatment that reduces illicit drug use, reduces the transmission of blood borne viruses (BBV), reduces mortality and improves mental and physical wellbeing [5, 9]. Engagement and retention in treatment improve outcomes with disruption in OAT (particularly on release from prison) associated with increased risk of overdose [9]. There are already many existing treatment deficits for opioid users which include inadequate access to OAT, needle and syringe exchange, naloxone programmes and overdose prevention advice [10]. Like other health care settings, drug treatment services will be presented with additional challenges during the COVID-19 pandemic. Priorities for addiction services include increasing access to OAT and maintaining continuity of care while protecting frontline staff [11].

Ireland like other European countries has a growing cohort of older drug users (over 40), mainly male, many who started heroin injecting in the late 1970s and early 1980s. Studies show that drug use exacerbates cardiovascular and respiratory disease and accelerates metabolic aging [7]. Older drug users are often more socially excluded, isolated from family, living in unstable accommodation and have higher levels of physical and mental health problems compared to their peers in the general population or younger drug users [7]. As COVID-19 disproportionally affects the older population and those with underlying conditions, this older drug users fall into an at-risk group.

Opioid use in Ireland is declining, particularly among younger drug users [12]. There are an estimated 18,000 opioid users in Ireland with over 10,000 on OAT [12, 13]. There is a large national network of specialist GPs providing OAT in addiction centres and in primary care. However, treatment deficits remain, particularly in smaller towns outside the larger urban centres where waiting times for OAT can be longer than 6 months [13]. People who use drugs (PWUD) who have been unable to access OAT to date are unlikely to follow public health advice if they are suffering withdrawal symptoms. It is therefore in the interest of both PWUD and society in general that they are provided with access to OAT as efficiently as possible.

During the COVID-19 crisis, healthcare is increasingly being delivered remotely [14, 15]. These models can reduce the risks to patients, healthcare staff and the public. This report describes a remote national model of OAT delivery that may inform other healthcare systems on delivering care to PWUD and others during these challenging times.

### Model development

Historically, a small number of OAT services have successfully used telemedicine for hepatitis c treatment and providing psychological and other supports [16–18]. Mental health services have also demonstrated success with remote care and of course general practice was one of the first medical specialities to adapt to its use to manage COVID-19 [14, 19].

In the immediate, expanding and adapting OAT services is critical to reduce the impact on COVID-19 and remote care models provide an opportunity to achieve this. These models may also have added benefits of OAT delivery into the future, potentially increasing access and improving retention in care [16]. How they are implemented and their impacts, both under normal and crises circumstances, inform how OAT services can adapt to manage the immediate and emerging risks of COVID and improve OAT delivery internationally.

Critically, the new remote model of OAT care was designed to minimise risk and to ensure compliance with accepted good practice for the treatment of opioid dependence. This model is underpinned by both national and international guidelines in particular with regard to assessment, diagnosis, supervision of medication and overdose risks [20–22].

In terms of pharmacological options to treat opiate dependence, substitution treatment using methadone is most common in Ireland, with buprenorphine-naloxone currently available on a limited basis [13]. Slow release formulations of OAT medications are not available in the Republic of Ireland (ROI) and despite their obvious potential were not considered for inclusion.

### Description

The Addiction Management in Primary Care Programme (AMPC) at the Irish College of General Practitioners developed a Standard Operation Procedure (SOP) that was endorsed by the HSE office for Social Inclusion and local managers nationally. Patients who are on existing waiting lists or who present seeking OAT during the current COVID-19 pandemic are included in this initiative. The following are details of the standard operating procedure for commencing a patient onto OAT using remote specialist consultation and prescribing:

Step 1: Addiction service nurse completes an up to date assessment via telephone with patient. Local drug treatment services will be funded to provide phones to patients who do not have telephone access. Following this telephone assessment, if the patient requests or the nurse considers he/she is suitable for OAT, the nurse will discuss this with the specialist prescribing doctors.

Step 2: Following a telephone triage to ensure patient does not have current COVID-related symptoms, the patient is given an appointment to attend the local addiction services to provide a point of care (POC) drug screen.

Patient must bring passport photo and photo identification to this appointment and sign central treatment list (CTL) entry form (the CTL is a national OAT register). If the patient is unable to provide photo identification, other formal identification will be accepted with a reasonable effort made by the local team to confirm its accuracy (e.g. home visit, confirmation by third party). The purpose of this step is to minimise the risk of double prescribing.

If POC result is not consistent with self-report, assessment process to be halted at this point and further advice/assessment to be sought from the prescriber. This is to reduce the risk of patients with low or no opioid tolerance being commenced on OAT.

Step 3: On completion of nurse assessment, drug screening and verification of ID—all documents to be healthmailed (healthmail is a secure electronic email system used in the ROI to share confidential medical information among health personnel) to remote specialist clinicians.

Step 4: CTL entry forms to be healthmailed by nurse.

Step 5: To fulfill Irish treatment regulations, pharmacy card will be issued and sent to pharmacy via healthmail and post. A copy to be healthmailed to prescribing doctor at the same time

Step 6: Video consultation arranged by prescriber and patient. Prior to prescribing, the specialist prescribing doctors will conduct a further assessment using an agreed standardised initial assessment tool.

Remote consultations will be guided by the HSE guidance document on remote consultation for addictions services [23].

Step 7: Agreed safe commencing prescribing protocols will be adhered to and methadone treatment will be commenced at 20 mg daily (or higher if deemed safe to do so by clinician). Subsequent doses can be titrated by 10 mg every 4 days following a telephone/video review with the patient. As pharmacy opening times and availability to provide daily supervision during the commencement phase may be compromised during the COVID-19 pandemic, due regard must be afforded by the prescriber for the safety of the patient and the safety of others.

Suboxone doses can be titrated in the normal fashion but will need more regular follow-up for the first week of induction. Patients will attend a local designated community pharmacy for daily supervision of their medication with take-aways limited to the days where the pharmacy is closed. This is a similar level of supervision to patients receiving face-to-care and consistent with national OAT guidelines [20].

Step 8: OAT script to be issued by the prescribing doctor directly to the pharmacy via secure e-prescribing method.

Step 9: Patient to be contacted by telephone daily/alternate days depending on individual risk factors by member of the local team (nurse or drugs worker) who has knowledge of the patient. During the induction phase, patients will be checked for intoxication, withdrawals and physical and psychological symptoms related to dosing. This will be done in consultation with the supervising community pharmacy who will be reviewing the patient on a daily basis. Each review will also include a check on patient management of medication, reported stability and the provision of psychological supports. If there are urgent concerns, nurse/drug worker will report these concerns to the prescriber.

Step 10: After the safe commencement of OAT, a weekly review will be conducted by the specialist prescribing doctor for ongoing scripting of OAT. If local services are not available for drug screening, reliance on self-report of drug use during the COVID-19 crisis will suffice. Self-reported drug use will be collected weekly and documented in the patient’s records. The results of local POC screens (limited) will also be updated to the patient’s electronic medical record and will be used in the final evaluation.

Step 11: A weekly virtual case review will take place between local services (senior nurse) and the specialist prescribers.

## Discussion

Rapid adaptation of OAT services is required to reduce the impact of COVID-19 on patients, staff and the general public [11]. Reducing the requirement for face-to-face consultations is recommended during this current pandemic [14]. In the case of addiction, treatment reducing the requirement to attend for on-site assessment, ongoing monitoring and provision of drug screens will increase the ability of patients to maintain social distance, self-isolate or cocoon if required. It also reduces the risk to frontline staff as an important consideration in ensuring continuity of services [3]. Remote care is particularly effective in rural areas because it reduces the need to travel and use of public transport.

While primary care was one of the first medical specialities to adapt to its use to manage COVID-19, a number of other specialities have reported on the use of novel remote models of care at this time [14, 15, 19]. Historically, OAT services have successfully used telemedicine for hepatitis C treatment and providing psychological and other supports [16–18, 24]. Mental health services have also demonstrated success with remote care [19].

It is recognised that OAT patients have high rates of psychological co-morbidities and managing anxiety and stress during this time will be problematic [25]. Coping with self-isolation, particularly if managing withdrawals, will be challenging. Many of the traditional face-to-face support networks for PWUD in recovery are no longer available. This model allows for the provision of remote psychological supports to the patient by a member of the local drug treatment service. While telephone consultations are adequate for most patients, video linking may be more appropriate for sicker patients and for those with greater psychosocial needs [14]. It is important to ensure that any system used to provide clinical care is secure, and patients’ data and medical information are protected and kept confidential. Furthermore, it is critical that patients’ medical records are updated to reflect the details of the remote care provided.

Induction onto OAT is recognised as a high-risk period for overdose and should be completed with caution [9]. Establishing the diagnosis of dependence and assessing tolerance is critical, as is OAT dosing over the first 4 days [9]. Supervision of OAT is recommended during induction and in the early stages of maintenance treatment [20–22]. Supervision improves compliance and reduces diversion, relapse to heroin and drug-related deaths (including those related to methadone) [26]. Prescribers may consider starting on lower methadone doses (e.g. 20 mg) to mitigate the risks associated with more rapid assessment, reduction in pre-treatment drug screening and reduced levels of supervision. Buprenorphine has a safer profile than methadone in induction but may be less successful in treatment retention [9]. During the COVID-19 pandemic, it is recognised that face to face pharmacy services may be reduced as well as more limited pharmacy opening hours. Access to daily direct supervision of consumption of OAT may therefore at times be unavailable to patients and assessment of increased risk will need to be addressed with patients. Further assessment of the impact of reduced supervision may provide services with an opportunity to re-evaluate this requirement post-COVID-19 as long-term pharmacy supervision is resource intensive and may promote dropout from treatment [26].

National and international guidelines recommend the use of drugs screening both in the diagnoses and monitoring of OAT [20–22]. There is little published evidence on the effectiveness of routine drug screening and screening schedules tend to be philosophical and historical within services [27]. This present challenge may give services an opportunity to evaluate the usefulness of drug screening and to implement a more evidence-based approach post-COVID-19.

While it is natural and critical to focus on emergency efforts to manage, treat and develop a vaccine to control this epidemic, it is important to evaluate new models of delivering medical and social care. These models will have been developed at a time of crisis but may have real benefits for patients and healthcare systems in the future. It is important that resources for these evaluations are factored into health budgets as many of these new measures may have significant health benefits and cost savings in a post-COVID future. This project includes an evaluation, outcomes of which will be reported at a later date.

## Conclusion

The threat caused by COVID-19 is challenging health systems globally. There is a requirement to adapt healthcare delivery to mitigate the risks. People who use opioids and other drugs have health and social risks that make them vulnerable during this COVID-19 epidemic. OAT services can adapt to reduce these risks, and these measures can have both immediate and future benefits to this group and inform how health and social care is delivered across our health services. Doctors and others working in the area need to advocate for the protection and even the expansion of harm reduction services and linked psychosocial supports during these challenging times. The Irish drug treatment services has developed a remote model for assessment and ongoing care of OAT patients that may inform other jurisdictions how to adapt care for opioid users and other both now and into the future.

## Data Availability

Not relevant as no data is being reported.

## Abbreviations

COVID-19: Coronavirus disease 2019
OAT: Opioid agonist therapy
WHO: World Health Organization
BBV: Blood-borne viruses
GPs: General practitioners
PWUD: People who use drugs
AMPC: Addiction management in primary care
SOP: Standard operation procedure
HSE: Health Service Executive
CTL: Central treatment list

## References

[1] WHO (2020). Situation Report-80 HIGHLIGHTS.

[2] Chen T, Wu D, Chen H, Yan W, Yang D, Chen G (2020). Clinical characteristics of 113 deceased patients with coronavirus disease 2019: retrospective study. BMJ.

[3] Lancet T. COVID-19: protecting health-care workers 2019. 10.1016/S0140-6736(20)30627-9.

[4] Cases in Ireland - Health Protection Surveillance Centre n.d. https://www.hpsc.ie/a-z/respiratory/coronavirus/novelcoronavirus/casesinireland/ (accessed 4 April 2020).

[5] Degenhardt L, Grebely J, Stone J, Hickman M, Vickerman P, Marshall BDL, et al. Global patterns of opioid use and dependence: harms to populations, interventions, and future action. Lancet 2019;394:1560–1579. 10.1016/S0140-6736(19)32229-9. 10.1016/S0140-6736(19)32229-9 PMC7068135 31657732

[6] Degenhardt L, Peacock A, Colledge S, Leung J, Grebely J, Vickerman P, et al. Global prevalence of injecting drug use and sociodemographic characteristics and prevalence of HIV, HBV, and HCV in people who inject drugs: a multistage systematic review. Lancet Glob Health 2017;5:e1192–e1207. 10.1016/S2214-109X(17)30375-3. 10.1016/S2214-109X(17)30375-3 PMC5683738 29074409

[7] Luxembourg: Publications Office for the European Union. European Monitoring Centre for Drugs and Drug Addiction. Treatment and care for older drug users. Selected issue 2010. Luxembourg. Luxemb Publ Off Eur Union 2010. 10.2810/39905.

[8] Degenhardt L, Charlson F, Stanaway J, Larney S, Alexander LT, Hickman M, et al. Estimating the burden of disease attributable to injecting drug use as a risk factor for HIV, hepatitis C, and hepatitis B: findings from the Global Burden of Disease Study 2013. Lancet Infect Dis 2016;16:1385–1398. 10.1016/S1473-3099(16)30325-5. 10.1016/S1473-3099(16)30325-5 27665254

[9] Sordo L, Barrio G, Bravo MJ, Indave BI, Degenhardt L, Wiessing L (2017). Mortality risk during and after opioid substitution treatment: systematic review and meta-analysis of cohort studies. BMJ.

[10] Larney S, Peacock A, Leung J, Colledge S, Hickman M, Vickerman P, et al. Global, regional, and country-level coverage of interventions to prevent and manage HIV and hepatitis C among people who inject drugs: a systematic review. Lancet Glob Health 2017;5:e1208–e1220. 10.1016/S2214-109X(17)30373-X. 10.1016/S2214-109X(17)30373-X PMC5683737 29074410

[11] EMCDDA update on the implications of COVID-19 for people who use drugs (PWUD) and drug service providers. n.d.

[12] Hay G, Jaddoa A, Oyston J, Webster J, Claire M, Hout V (2017). Estimating the prevalence of problematic opiate use in ireland using indirect statistical methods.

[13] Delargy I, Crowley D, Van Hout MC (2019). Twenty years of the methadone treatment protocol in Ireland: reflections on the role of general practice. Harm Reduct J.

[14] Greenhalgh T, Choon Huat Koh G, Car J. Covid-19: a remote assessment in primary care n.d. 10.1136/bmj.m1182. 10.1136/bmj.m1182 32213507

[15] Kirby T. Rheumatologists rapidly adjust patient care during COVID-19 pandemic n.d. 10.1016/S2665-9913(20)30094-1. 10.1016/S2665-9913(20)30094-1 PMC7270630 32518921

[16] Eibl JK, Gauthier G, Pellegrini D, Daiter J, Varenbut M, Hogenbirk JC (2017). The effectiveness of telemedicine-delivered opioid agonist therapy in a supervised clinical setting. Drug Alcohol Depend.

[17] Marciano S, Haddad L, Plazzotta F, Mauro E, Terraza S, Arora S (2017). Implementation of the ECHO ®telementoring model for the treatment of patients with hepatitis C. J Med Virol.

[18] Allison LL, Casteel D, Shigekawa E, Weyrich MS, Roby DH, McMenamin SB (2019). Telemedicine-delivered treatment interventions for substance use disorders: A systematic review. J Subst Abus Treat.

[19] Zhou X, Snoswell CL, Harding LE, Bambling M, Edirippulige S, Bai X, et al. The Role of Telehealth in Reducing the Mental Health Burden from COVID-19. Telemed e-Health 2020:tmj.2020.0068. 10.1089/tmj.2020.0068. 10.1089/tmj.2020.0068 32202977

[20] Health Service Executive. Clinical guidelines for opioid substitution treatment. Dublin: 2017.

[21] of Health D. Drug misuse and dependence UK guidelines on clinical management. n.d.

[22] New South Wales. Ministry of Health. NSW clinical guidelines : treatment of opioid dependence - 2018. n.d.

[23] HSE National Covid 19 Telehealth Steering Committee. Clinical governance guidance on secure video and audio consultations during the emergency measures to address Covid 19. Dublin: 2020.

[24] Marsch LA, Guarino H, Acosta M, Aponte-Melendez Y, Cleland C, Grabinski M (2014). Web-based behavioral treatment for substance use disorders as a partial replacement of standard methadone maintenance treatment. J Subst Abus Treat.

[25] Brooner RK, King VL, Kidorf M, Schmidt CW, Bigelow GE (1997). Psychiatric and substance use comorbidity among treatment-seeking opioid abusers. Arch Gen Psychiatry.

[26] Cousins G, Boland F, Barry J, Lyons S, Keenan E, O’Driscoll D (2017). J-shaped relationship between supervised methadone consumption and retention in methadone maintenance treatment (MMT) in primary care: National cohort study. Drug Alcohol Depend.

[27] McEachern J, Adye-White L, Priest KC, Moss E, Gorfinkel L, Wood E (2019). Lacking evidence for the association between frequent urine drug screening and health outcomes of persons on opioid agonist therapy. Int J Drug Policy.

